# Development of an organ‐specific insert phantom generated using a 3D printer for investigations of cardiac computed tomography protocols

**DOI:** 10.1002/jmrs.279

**Published:** 2018-04-30

**Authors:** Kamarul A. Abdullah, Mark F. McEntee, Warren Reed, Peter L. Kench

**Affiliations:** ^1^ Discipline of Medical Radiation Sciences Faculty of Health Sciences The University of Sydney Lidcombe New South Wales Australia; ^2^ Faculty of Health Sciences Universiti Sultan Zainal Abidin Terengganu Malaysia

**Keywords:** 3D printing, cardiac insert phantom, computed tomography, computer aided design (CAD), rapid prototyping

## Abstract

**Introduction:**

An ideal organ‐specific insert phantom should be able to simulate the anatomical features with appropriate appearances in the resultant computed tomography (CT) images. This study investigated a 3D printing technology to develop a novel and cost‐effective cardiac insert phantom derived from volumetric CT image datasets of anthropomorphic chest phantom.

**Methods:**

Cardiac insert volumes were segmented from CT image datasets, derived from an anthropomorphic chest phantom of Lungman N‐01 (Kyoto Kagaku, Japan). These segmented datasets were converted to a virtual 3D‐isosurface of heart‐shaped shell, while two other removable inserts were included using computer‐aided design (CAD) software program. This newly designed cardiac insert phantom was later printed by using a fused deposition modelling (FDM) process via a Creatbot DM Plus 3D printer. Then, several selected filling materials, such as contrast media, oil, water and jelly, were loaded into designated spaces in the 3D‐printed phantom. The 3D‐printed cardiac insert phantom was positioned within the anthropomorphic chest phantom and 30 repeated CT acquisitions performed using a multi‐detector scanner at 120‐kVp tube potential. Attenuation (Hounsfield Unit, HU) values were measured and compared to the image datasets of real‐patient and Catphan^®^ 500 phantom.

**Results:**

The output of the 3D‐printed cardiac insert phantom was a solid acrylic plastic material, which was strong, light in weight and cost‐effective. HU values of the filling materials were comparable to the image datasets of real‐patient and Catphan^®^ 500 phantom.

**Conclusions:**

A novel and cost‐effective cardiac insert phantom for anthropomorphic chest phantom was developed using volumetric CT image datasets with a 3D printer. Hence, this suggested the printing methodology could be applied to generate other phantoms for CT imaging studies.

## Introduction

Over the past few years, there has been increased use of 3D printing technology for rapid prototyping of high‐quality printed objects.^1^ Since its introduction, 3D printing technology has been successfully applied in numerous areas, such as engineering, industry, art, education and medicine.^2^, ^3^ In medicine, 3D printing technology has been used for a variety of purposes, for example, assisting surgical planning,^4^ guiding interventional procedures,^5^ manufacturing radiology components,^6^ printing personalised artificial parts^7^ and recently, developing phantoms.^8^, ^9^, ^10^, ^11^

Phantoms have been widely applied in medical imaging, especially in CT systems, commonly for both quantitative and qualitative assessments of image quality. Many prior studies^12^, ^13^, ^14^ have highlighted the advantages of using phantoms, especially when the investigations involve multiple radiation exposures with different acquisitions settings. One of the most common phantoms used for the investigations of CT protocols is the anthropomorphic chest phantom (Kyoto Kagaku, Japan). This phantom has properties that are very similar to the anatomical features of an adult chest region, for example, the lungs, bones and muscles. However, the cardiac insert of this phantom has single, homogenous material that is not appropriate to simulate CT images, especially for cardiac CT. An ideal cardiac insert should be able at least to simulate the heart features with appropriate appearances in the resultant CT images.

Recently, many recent phantom studies^8^, ^9^, ^10^, ^11^, ^15^, ^16^ have employed the 3D printing technology to construct their phantoms. For example, Solomon et al.,^16^ asserted that this technology could be applied to generate anthropomorphic texture phantoms that are feasible to assess the quality of CT images. Another was by Shuai Leng et al.,^15^ which used 3D printing technology to generate a comprehensive quality assurance phantom. However, the major drivers are the limitations of available commercial phantoms which are often costly and not customisable. This 3D printing technology allows researchers to design and construct physical phantoms and organ inserts based on their preferences at a lower cost than any commercially available physical phantoms. Additionally, the successful and validated 3D‐printed physical phantoms can be reproduced by any other accessible 3D printers.

Therefore, it is indeed possible to fabricate 3D‐printed phantoms with specific characteristics to suit various imaging investigations, particularly in CT systems. In this study, the investigation of 3D printing technology offers an alternative to produce a novel and cost‐effective cardiac insert phantom containing a contrast‐enhanced region directly from volumetric CT image datasets of anthropomorphic chest phantom. The printing methodology used in this study could be generally applied to generate other phantoms for CT imaging studies.

## Materials and Methods

The following three steps were taken to develop the new cardiac insert phantom:

1. **Step one** involved obtaining acquisitions of volumetric datasets from a multi‐detector CT scanner.
2. **Step two** involved delineating the regions of interest (ROI) from the surrounding structures, which resulted in segmented image datasets. This step also included the optimisation procedure, such as smoothing and wrapping.
3. **Step three** involved printing the new physical phantom and removing unnecessary supporting structures so as to produce at final clean physical 3D‐printed cardiac insert phantom.

The phantom was printed using a 3D printer of Creatbot DM Plus Model (Mankati, Shanghai, China). This 3D printer uses a fused deposition modeling (FDM) technique to develop the phantom. This FDM technique is similar to inkjet printing but a filament is used instead of ink. Acrylonitrile butadiene styrene (ABS) filament passes through a moving heated extruder to print ~0.25 mm layer of material onto the build tray. Next, a cooling fan solidifies the ABS material creating a traced layer onto the tray. The process was then repeated for each layer until completed. Additional support materials were also printed on the layers to prevent the structures from collapsing.

The proceeding sections elaborate on the (1) designs of the 3D‐printed cardiac insert phantom, (2) process of printing the physical models, (3) after‐printing process and (4) measurement of attenuation (HU) values.

### Phantom design

**Figure 1:**
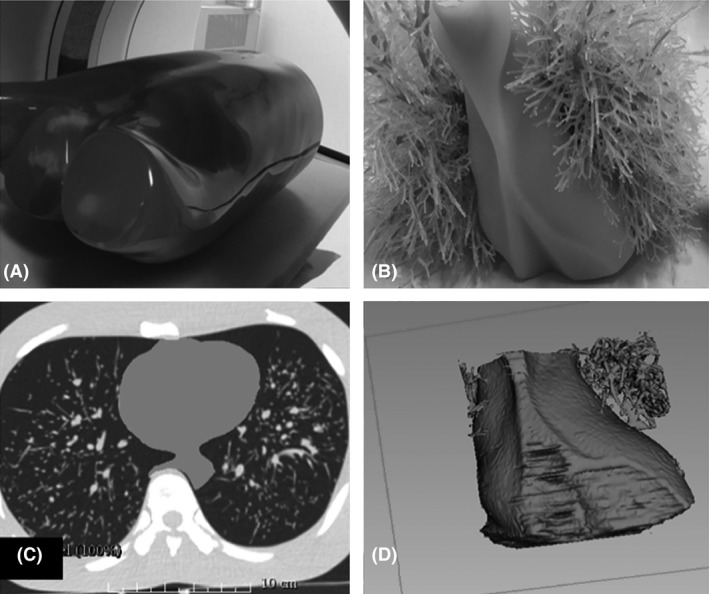
(A) An anthropomorphic chest phantom (Lungman N‐01, Kyoto Kagaku, Co., Ltd., Kyoto, Japan). The anthropomorphic chest phantom was scanned on a multi‐detector CT scanner in order to obtain the volumetric datasets of the original cardiac insert; (B) The original size and the appearance of the cardiac insert; (C) The segmentation process using 3D Slicer software program (The Slicer Community, Harvard).^17^ The cardiac insert was segmented to ensure that the modelling process could be performed to produce the heart‐shaped shell; and (D) The virtual 3D model of the original cardiac insert.
- The removable inserts were designed to have similar structures as coronary arteries or ascending aorta (A) and ventricular anatomy (B) and fit within the heart‐shaped shell. Insert A was designed with varying diameters of cylindrical structures in order to resemble the different sizes of contrast‐enhanced regions of the coronary arteries and ascending aorta placed on both sides of the insert to represent the right and left sides of the heart. The diameters of the coronary arteries and the ascending aorta were set from 1.5 to 5.0 mm and 30 mm, respectively. A minimum diameter of 1.5 mm is the smallest diameter detectable as in accordance to the American Heart Association (AHA) Guidelines.^18^ The dimensional size of these two removable inserts was further adjusted so that they fitted and could be suitably positioned in the heart‐shaped shell. Figure 2A–C shows the cross‐sectional diagram and the virtual 3D‐isosurface of the removable inserts A and B.

The 3D‐printed cardiac insert phantom was made of two main assemblies, (i) the heart‐shaped shell and (ii) the removable inserts:

- The heart‐shaped shell design was derived from the volumetric CT image datasets of an anthropomorphic chest phantom (Lungman N‐01, Japan; see Fig. 1A–B). The cardiac insert was scanned on a multi‐detector CT scanner (Alexion, Toshiba Medical Systems Co Ltd., Otowara, Japan) using a 120‐kVp tube potential and a fixed 200 mA tube current. The reconstructed image datasets were transferred to a segmentation software program (3D Slicer, The Slicer Community, Harvard)^17^ to delineate and outline structures from the carina to the apex of the heart phantom, see Figure 1C–D. Next, the segmented image datasets were exported to generate a virtual 3D‐isosurface of the cardiac insert using Rhinoceros 3D (McNeel, Seattle, WA, USA) and Autodesk 123D Design (Autodesk Inc., San Rafael, CA, USA) software. To remove any defect, smoothing and surface rendering methods were performed on the 3D‐surface mesh. The mesh of the cardiac insert was then saved in a binary stereolithography (STL) file format before it was exported to the 3D printer for printing. The heart‐shaped shell was designed to ensure it could be suitably positioned in the anthropomorphic chest phantom for CT scanning.

**Figure 2:**
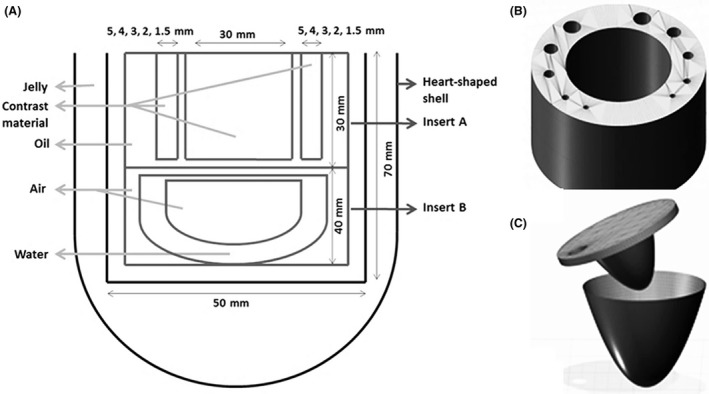
(A) A cross‐sectional diagram of the new custom‐made design of 3D‐printed cardiac insert phantom. The measurements of each model were determined based on the adjustments made so that the model could fit the size of the heart‐shaped shell perfectly, as well as to be suitably positioned in the anthropomorphic chest phantom. The modelling parts of the removable inserts within the heart‐shaped shell are (B) removable insert A, and (C) removable insert B.

### Printing process

**Figure 3:**
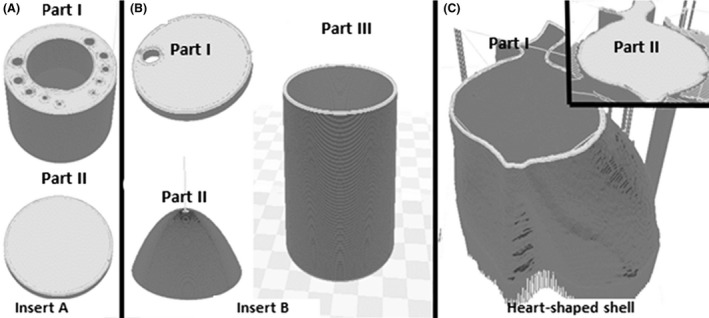
Three separate tasks were carried out to facilitate the printing tasks. (A) Insert A was divided into Parts I and II; (B) Insert B was separated into three parts (Parts I, II and III); and (C) Heart‐shaped shell was divided into Parts I and II. These separation tasks of printing parts eased the process of filling with varied density materials after the printing process.

Three printing tasks had been employed to facilitate the printing process, which were: (i) Insert A; (ii) Insert B; and (iii) Heart‐shaped shell. Insert A was printed by segregating it into Parts I and II. Part I refers to the cylindrical structures, while Part II denotes the base layer (Fig. 3A). Meanwhile, Insert B was divided into three parts (Parts I, II and III). Part I refers to the top layer, while Part II denotes the ventricle‐shape and Part III reflects the outermost cylinder shape in which to insert both the removable inserts (Fig. 3B). As for the heart‐shaped shell, it was separated into Parts I and II, where Part I is for the shell where the removable inserts could be placed, whereas Part II is the top layer (Fig. 3C). Such division of printing parts or assemblies allowed easy filling for the varied density materials, especially after the printing process. The printer settings used during the 3D printing process were configured based on the Simplify3D (Ohio, USA) software program, as shown in Table 1.

**Table 1:** The 3D printer settings applied in this study. In achieving very fine details with several ranges of printing materials or 3D printer while avoiding gaps, leaking and overlaps; varying results could be generated. NB These settings are only applicable if a printer similar to Creatbot DM Plus Model (Mankati, Shanghai, China) and a software program similar to Simplify 3D (Ohio, USA) are employed to design and to construct the phantom

Settings	Selection
i. Extruder toolhead	Nozzle diameter: 0.40 mm, Extrusion multiplier: 1.00, Extrusion width: Auto, Retraction distance: 1.00 mm, Retraction speed: 1800.0 mm/min
ii. Layer	Primary layer height: 0.25 mm, Top/bottom solid layers: 5, Outline/perimeter shells: 5, Outline direction: Inside‐out, First layer height: 90%, First layer width: 100%, First layer speed: 50%
iii. Additions: Raft	Raft layers: 1, Raft offset from part: 2.00 mm, Separation distance: 1.50 mm, Raft infill: 100%
iv. Infill	Internal fill pattern: Grid, External fill pattern: Concentric, Interior fill percentage: 10%, Outline overlap: 50%, Minimum infill length: 5.00 mm, Print sparse infill every: 1 layer, Infill angle offsets: 45/−45°
v. Support: Generate support material	Support infill percentage: 25%, Dense support layers: 5 Dense infill percentage: 50%, Print support every layer Support type: Normal, Support pillar resolution: 4.00 mm
vi. Temperature	Extruder: 240°C Heated Bed/Platform: 230°C
vii. Cooling	Fan speed: 60%
viii. G‐code	Tick all boxes: 5D firmware, allow zeroing of extrusion distance, firmware supports ‘sticky’ parameters, update machine definition (Cartesian robot), update firmware configuration (Rep/Rap)
ix. Script	G28; home all axes
x. Others	Default printing speed: 1800.0 mm/min Outline under speed: 50% Solid infill under speed: 80% X/Y axis movement speed: 4800.0 mm/min Z axis movement speed: 1000.0 mm/min Filament diameter: 1.7500 mm

### After‐printing process

Additional support materials, for example, rafts and pillars, were removed from the 3D‐printed cardiac insert phantom. Next, the external surface of heart‐shaped shell and removable inserts (Inserts A and B) was covered with acrylonitrile butadiene styrene (ABS) liquid to prevent leakage of the materials. This ABS liquid was produced by soaking the ABS filaments into acetone for approximately 30–45 min. All the removable inserts were glued together after the process of filling the phantom with materials of different densities was completed. The heart‐shaped shell that supported the two inserts was then filled with jelly to simulate the myocardium. Insert A was filled with oil, while the surrounding tube‐like structures were filled with Ultravist‐370 (Schering Health Care Ltd, Burgess Hill, UK) iodinated contrast media to resemble the contrast‐enhanced vessels. The iodine concentration was adjusted to simulate cardiac CT imaging of coronary CT angiography at 100–120 kVp, 25–30 HU per mg of iodine per ml.^19^ Insert B was filled with water material and separately with air material, where the latter simulated the trachea.

### Attenuation properties

The average attenuation (Hounsfield Unit, HU) values were measured to verify the properties of the phantom for cardiac CT imaging. All measurements were performed at the CT scanner workstation. The 3D‐printed cardiac insert phantom was positioned in the anthropomorphic chest phantom and imaged 30 times with a multi‐detector CT scanner (Alexion, Toshiba Medical Systems). The acquisitions of the phantom were performed at 120‐kVp tube potential, scan FOV 250 mm and 0.75‐s rotation time. The tube current was set at 200 mA and dose modulation was turned off. The projection image datasets were reconstructed by applying only filtered back projection (FBP) and FC18 reconstruction kernel with a 1.0‐mm slice thickness and an axial FOV of 160‐mm. The average HU values were measured by placing the ROI over each material (contrast media, air, oil, and jelly) reconstructed axial images of the cardiac phantom, the relevant anatomy (ascending aorta, air, fat and muscle) of patient image datasets and also the air and LDPE inserts of Catphan^®^ 500 phantom (The Phantom Laboratory, Salem NY, USA). Both patient and Catphan^®^ 500 phantom datasets were scanned at similar acquisition protocols.

## Results

**Figure 4:**
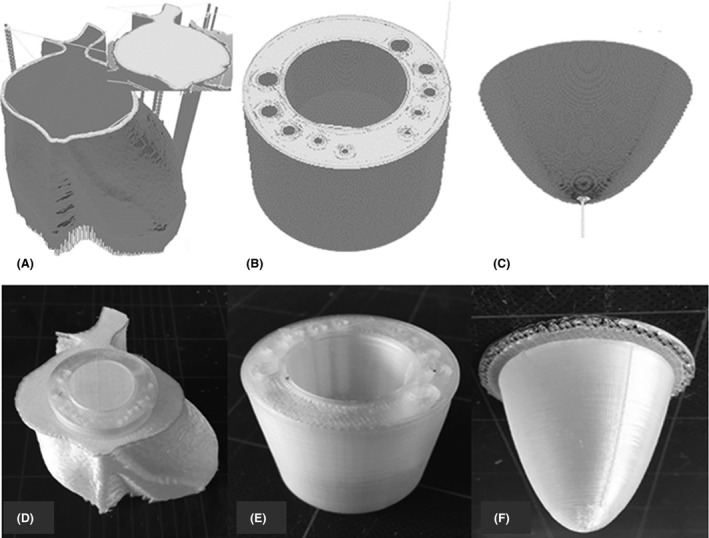
A 3D‐printed cardiac insert phantom; heart‐shaped shell, insert A, and insert B, before (A–C) and after the printing process (D–F) respectively.

**Figure 5:**
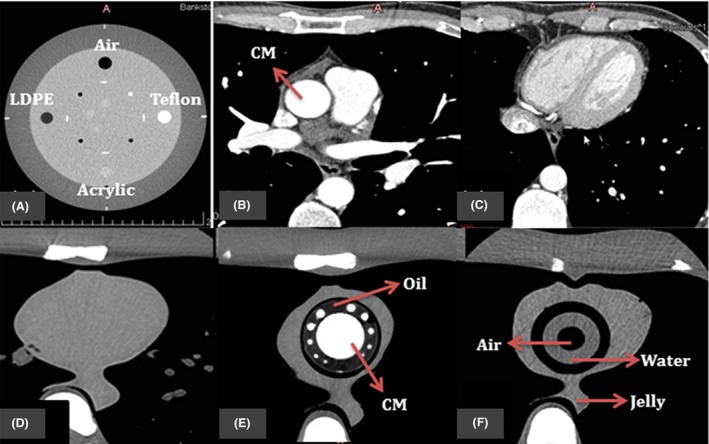
The resulting axial CT images of (A) four inserts in Catphan® 500 phantom; (B) and (C) patient image datasets for cardiac CT; (D) original cardiac insert of anthropomorphic chest phantom; (E–F) 3D‐printed cardiac insert phantom with contrast materials (CM), oil, air, water and jelly segments labelled.

The physical models and the axial CT images of the completed 3D‐printed cardiac insert phantom are illustrated in Figures 4 and 5 respectively. The total printing time was 12.1 hours and phantom preparation time, for example, removing support materials, covering surfaces with ABS liquid, assembling all parts and filling the phantom with materials was 10.2 hours. The cost of the phantom production was approximately US$70, which covered the costs of the ABS filament and the internal materials used. However, the cost of the 3D printer was excluded due to institute ownership.

The mean attenuation (HU) values for circular ROI placed over varied materials within 3D‐printed cardiac insert phantom, real‐patient image datasets, and Catphan^®^ 500 are tabulated in Table 2 for FBP image reconstruction algorithms. As a result, the measured values confirmed that the materials used in the 3D‐printed cardiac insert phantom are comparable with those obtained from real‐patient image datasets and the standard CT image quality phantom Catphan^®^ 500 phantom (Air and LDPE inserts).

**Table 2:** Mean of attenuation (HU) values obtained with FBP (FC18) for the 3D‐printed cardiac insert, as compared to the patient image datasets, and Catphan^®^ 500 at 120 kVp

HU	Contrast material	Air	Oil/Fat	Jelly/Muscle
3D‐printed cardiac insert	354.3	−894.1	−92.4	25.9
Patient image datasets	327.0	−847.5	−90.0	17.6
Catphan^®^ 500	n/a	−968.9	−83.0	n/a

n/a, not available.

## Discussion

This paper presents a novel design of a 3D‐printed cardiac insert phantom for an anthropomorphic chest phantom, including the associated 3D printing methodology. This phantom was comprised of a contrast‐enhanced region to enable the investigation of the impact of various settings upon cardiac CT protocols. In a prior work,^20^ the use of this new cardiac insert phantom had been demonstrated to determine the impact of various image reconstruction algorithms on image quality and dose reduction potential. The results were consistent with past studies^21^, ^22^, ^23^ as the image datasets reconstructed with iterative reconstruction algorithm exhibited more noise reduction, hence resulting in higher image quality, when compared to the filtered back projection.

To ascertain image quality, researchers^24^, ^25^, ^26^, ^27^ measured image noise by placing the ROI within a specific anatomical contrast‐enhanced region to ascertain image quality. For cardiac CT imaging of coronary CT angiography, the ROI is usually placed within the ascending aorta.^24^, ^25^, ^26^ In clinical case, this anatomical region refers to the time‐to‐peak enhancement of the contrast media, which has been often applied to test the adequacy of the contrast path, and therefore, overall contrast enhancement level as well as diagnostic image quality.^28^ For the 3D‐printed cardiac insert phantom, a cylindrical contrast‐enhanced region was designed with similar diameter to the average ascending aorta (~30 mm). The large size of this cylindrical contrast‐enhanced region allowed for the measurement of image noise.

Despite image noise, most clinical‐based studies^29^, ^30^, ^31^ also employed the detectability of coronary arteries to determine the subjective image quality that resulted from varying protocols. For instance, Carrascosa et al.,^30^ determined the overall image quality score based on coronary artery visualisation. As for the present cardiac insert phantom, the varying size of cylindrical contrast‐enhanced regions represented this purpose. Hence, the edges and the detectability of these cylindrical contrast‐enhanced regions over various protocols applied could be used to determine the overall subjective image quality.

Another advantage of this new insert phantom is the removable inserts. This new feature allowed the researchers to further customise the design or the filling materials used to suit their purposes. Additionally, this design was successfully developed by using a CAD software program, hence making it possible for other researchers to redesign and reproduce new physical phantom models. In fact, numerous other open sources software programs are also available on the internet for users to download and use to build their phantom designs.

The primary challenge of 3D printing had been seeking the most apt printing methodology, which is inclusive of selecting suitable printing materials, determining the correct temperature settings of the extruders, and choosing the most appropriate printer protocols.^32^ From this work of developing the present phantom, deciding on the appropriate temperature for the extruder to lay the printing materials on the platform had been an intricate issue. Another problem that was experienced had been during the printing process of the removable inserts due to the surface intricacy and the size of subtle diameters.

The new insert phantom offers a good alternative to researchers who need to produce custom phantoms relatively quickly and cost‐effectively. Sophisticated phantom production demands the use of the latest 3D printer technology that allows a greater variety of filament materials and the ability to customise phantoms with the desired geometrical features. There are several limitations related to the 3D printer used. First, the new insert phantom resembled a static physical model of a dynamic organ meaning the various changes that take place during the cardiac cycle were not displayed in the projection images. Different printing materials moved by electrical motors could offer a sequence of mechanical events similar to heartbeats. Second, more advanced 3D printing technology can produce physical models with highly intricate surfaces and sides. This advanced technology could be extended to produce a phantom from the real‐patient volumetric CT datasets.

This 3D‐printed cardiac insert phantom was also comparable with the HU values obtained from the real‐patient image datasets and the Catphan^®^ 500 phantom. Hence, it was likely that for all the filling materials, the resulting image quality assessments did display similar results upon using the real‐patients or Catphan^®^ 500 phantom. Nonetheless, in any case, additional investigations, for example, resolutions and detectability, using other tools are indeed necessary to ensure that the image quality assessments are accurate.

## Conclusions

In conclusion, this study demonstrates that a novel 3D‐printed cardiac insert phantom can be produced from volumetric CT images. This new insert phantom could be reproduced by investigators who need a relatively cost‐ and time‐effective method of producing customised CT phantoms. Further advances in this 3D printing technology promise to offer more flexibility in design, and this could become a more routine method in producing phantoms in future.

## Conflict of Interest

The authors declare no conflict of interest.

## References

[1] Oropallo W , Piegl LA . Ten challenges in 3D printing. Engineering with Computers 2015; 32: 135–48.

[2] Leng S , Chen B , Vrieze T , et al. Construction of realistic phantoms from patient images and a commercial three‐dimensional printer. J Med Imaging (Bellingham) 2016; 3: 033501. 27429998 10.1117/1.JMI.3.3.033501 PMC4935810

[3] Michalski MH , Ross JS , Practice M . The shape of things to come: 3D printing in medicine. Am Med Assoc 2014; 312: 2213–4. 10.1001/jama.2014.9542 25461994

[4] Giannopoulos AA , Mitsouras D , Yoo SJ , Liu PP , Chatzizisis YS , Rybicki FJ . Applications of 3D printing in cardiovascular diseases. Nat Rev Cardiol 2016; 13: 701–18. 27786234 10.1038/nrcardio.2016.170

[5] Ebert LC , Thali MJ , Ross S . Getting in touch–3D printing in forensic imaging. Forensic Sci Int 2011; 211: e1–6. 21602004 10.1016/j.forsciint.2011.04.022

[6] Miller BW , Moore JW , Barrett HH , et al. 3D printing in x‐ray and gamma‐ray imaging: A novel method for fabricating high‐density imaging apertures. Nucl Instrum Methods Phys Res A 2011; 659: 262–8. 22199414 10.1016/j.nima.2011.08.051 PMC3244175

[7] Goyanes A , Det‐Amornrat U , Wang J , Basit AW , Gaisford S . 3D scanning and 3D printing as innovative technologies for fabricating personalized topical drug delivery systems. J Control Release 2016; 234: 41–8. 27189134 10.1016/j.jconrel.2016.05.034

[8] Whiting BR , Hoeschen C , Solomon J , Bochud F , Samei E . Design of anthropomorphic textured phantoms for CT performance evaluation. Medical Imaging 2014: Physics of Medical Imaging; 2014.

[9] Leng S , Yu L , Vrieze T , Kuhlmann J , Chen B , McCollough CH . Construction of realistic liver phantoms from patient images using 3D printer and its application in CT image quality assessment. Proc SPIE Int Soc Opt Eng 2015; 2015: 94124E. 10.1117/12.2082121 PMC5051578 27721555

[10] Madamesila J , McGeachy P , Villarreal Barajas JE , Khan R . Characterizing 3D printing in the fabrication of variable density phantoms for quality assurance of radiotherapy. Phys Med 2016; 32: 242–7. 26508016 10.1016/j.ejmp.2015.09.013

[11] Kim MJ , Lee SR , Lee MY , et al. Characterization of 3D printing techniques: Toward patient specific quality assurance spine‐shaped phantom for stereotactic body radiation therapy. PLoS ONE 2017; 12: e0176227. 28472175 10.1371/journal.pone.0176227 PMC5417437

[12] Aurumskjold ML , Ydstrom K , Tingberg A , Soderberg M . Improvements to image quality using hybrid and model‐based iterative reconstructions: A phantom study. Acta Radiol 2017; 58: 53–61. 26924832 10.1177/0284185116631180

[13] Baek JH , Lee W , Chang KH , Chung JW , Park JH . Optimization of the scan protocol for the reduction of diaphragmatic motion artifacts depicted on CT angiography: A phantom study simulating pediatric patients with free breathing. Korean J Radiol 2009; 10: 260–8. 19412514 10.3348/kjr.2009.10.3.260 PMC2672181

[14] Ghetti C , Ortenzia O , Serreli G . CT iterative reconstruction in image space: A phantom study. Phys Med 2012; 28: 161–5. 21497530 10.1016/j.ejmp.2011.03.003

[15] Leng S , McGee K , Morris J , et al. Anatomic modeling using 3D printing: Quality assurance and optimization. 3D Print Med 2017; 3: 1–14. 29782614 10.1186/s41205-017-0014-3 PMC5954797

[16] Solomon J , Samei E . Quantum noise properties of CT images with anatomical textured backgrounds across reconstruction algorithms: FBP and SAFIRE. Med Phys 2014; 41: 091908. 25186395 10.1118/1.4893497

[17] Fedorov A , Beichel R , Kalpathy‐Cramer J , et al. 3D Slicer as an image computing platform for the Quantitative Imaging Network. Magn Reson Imaging 2012; 30: 1323–41. 22770690 10.1016/j.mri.2012.05.001 PMC3466397

[18] Austen WG . A reporting system on patients evaluated for coronary artery disease. Circulation 1975; 51: 5–40. 1116248 10.1161/01.cir.51.4.5

[19] Bae KT . Intravenous contrast medium administration and scan timing at CT: Considerations and approaches. Radiology 2010; 256: 32–61. 20574084 10.1148/radiol.10090908

[20] Abdullah KA , McEntee MF , Reed W , Kench PL . Using 3D printed cardiac CT phantom for dose reduction and diagnostic image quality assessment. J Med Radiat Sci 2017; 64: 93–105.

[21] Abdullah KA , McEntee MF , Reed W , Kench PL . Radiation dose and diagnostic image quality associated with iterative reconstruction in coronary CT angiography: A systematic review. J Med Imaging Radiat Oncol 2016; 60: 459–68. 27241506 10.1111/1754-9485.12473

[22] Willemink MJ , Takx RA , de Jong PA , et al. Computed tomography radiation dose reduction: Effect of different iterative reconstruction algorithms on image quality. J Comput Assist Tomogr 2014; 00: 1–9. 10.1097/RCT.0000000000000128 24983438

[23] Guariglia S , Meliadò G , Zivelonghi E , Pinali L , Montemezzi S , Cavedon C . Dose reduction and image quality in CT examinations using an iterative reconstruction algorithm: A phantom study. Biomed Phys Eng Express 2015; 1: 045203.

[24] Park EA , Lee W , Kim KW , et al. Iterative reconstruction of dual‐source coronary CT angiography: Assessment of image quality and radiation dose. Int J Cardiovasc Imaging 2012; 28: 1775–86. 22187198 10.1007/s10554-011-0004-2

[25] Tatsugami F , Matsuki M , Nakai G , et al. The effect of adaptive iterative dose reduction on image quality in 320‐detector row CT coronary angiography. Br J Radiol 2012; 85: e378–82. 22253355 10.1259/bjr/10084599 PMC3495581

[26] Wang R , Schoepf UJ , Wu R , et al. CT coronary angiography: Image quality with sinogram‐affirmed iterative reconstruction compared with filtered back‐projection. Clin Radiol 2013; 68: 272–8. 22981731 10.1016/j.crad.2012.08.007

[27] Yoo RE , Park EA , Lee W , et al. Image quality of adaptive iterative dose reduction 3D of coronary CT angiography of 640‐slice CT: Comparison with filtered back‐projection. Int J Cardiovasc Imaging 2013; 29: 669–76. 22923280 10.1007/s10554-012-0113-6

[28] Cademartiri F , Nieman K , van der Lugt A , et al. Intravenous contrast material administration at 16‐detector row helical CT coronary angiography: Test bolus versus bolus‐tracking technique. Radiology 2004; 233: 817–23. 15516601 10.1148/radiol.2333030668

[29] Utsunomiya D , Weigold WG , Weissman G , Taylor AJ . Effect of hybrid iterative reconstruction technique on quantitative and qualitative image analysis at 256‐slice prospective gating cardiac CT. Eur Radiol 2012; 22: 1287–94. 22200900 10.1007/s00330-011-2361-6

[30] Carrascosa P , Rodriguez‐Granillo GA , Capunay C , Deviggiano A . Low‐dose CT coronary angiography using iterative reconstruction with a 256‐slice CT scanner. World J Cardiol 2013; 5: 382–6. 24198908 10.4330/wjc.v5.i10.382 PMC3817280

[31] Fuchs TA , Stehli J , Bull S , et al. Coronary computed tomography angiography with model‐based iterative reconstruction using a radiation exposure similar to chest X‐ray examination. Eur Heart J 2014; 35: 1131–6. 24553723 10.1093/eurheartj/ehu053 PMC4006092

[32] Ionita CN , Mokin M , Varble N , et al. Challenges and limitations of patient‐specific vascular phantom fabrication using 3D Polyjet printing. Proc SPIE Int Soc Opt Eng 2014; 9038: 90380M. 10.1117/12.2042266 PMC4188370 25300886

